# Reinforcement learning algorithm for improving speed response of a five-phase permanent magnet synchronous motor based model predictive control

**DOI:** 10.1371/journal.pone.0316326

**Published:** 2025-01-03

**Authors:** Ahmed M. Hassan, Jafar Ababneh, Hani Attar, Tamer Shamseldin, Ahmed Abdelbaset, Mohamed Eladly Metwally

**Affiliations:** 1 Department of Electrical Power and Machines Engineering, Faculty of Engineering, Benha University, Shoubra, Cairo, Egypt; 2 Department of Electrical Power and Machines Engineering, Higher Institute of Engineering (HIE), El-Shorouk Academy, El-Shorouk City, Egypt; 3 Cyber Security Department, Faculty of Information Technology, Zarqa University, Zarqa, Jordan; 4 Faculty of Engineering, Zarqa University, Zarqa, Jordan; 5 College of Engineering, University of Business and Technology, Jeddah, Saudi Arabia; 6 Technical Research Center, Cairo, Egypt; National University of Computer and Emerging Sciences - Lahore Campus, PAKISTAN

## Abstract

Enhancing the performance of 5ph-IPMSM control plays a crucial role in advancing various innovative applications such as electric vehicles. This paper proposes a new reinforcement learning (RL) control algorithm based twin-delayed deep deterministic policy gradient (TD3) algorithm to tune two cascaded PI controllers in a five-phase interior permanent magnet synchronous motor (5ph-IPMSM) drive system based model predictive control (MPC). The main purpose of the control methodology is to optimize the 5ph-IPMSM speed response either in constant torque region or constant power region. The speed responses obtained using RL control algorithm are compared with those obtained using four of the most recent metaheuristic optimization techniques (MHOT) which are Transit Search (TS), Honey Badger Algorithm (HBA), Dwarf Mongoose (DM), and Dandelion-Optimizer (DO) optimization techniques. The speed response are compared in terms of the settling time, rise time, maximum time and maximum overshoot percentage. It is found that the suggested RL based TD3 give minimum settling time and relatively low values for the rise time, max time and overshoot percentage which makes the RL provide superior speed responses compared with those obtained from the four MHOT. The drive system speed responses are obtained in the constant torque region and constant power region using MATLAB SIMULINK package.

## 1. Introduction

IPMSMs are highly suitable for high-performance drive systems due to their various advantages. These include exceptional performance, high efficiency, compact size, low noise, and reliability [1–3]. IPMSMs are widely used in various applications, with robots and electric vehicles being among the most common [2, 3]. Due to their inveterate advantages, multiphase motor drives are gaining prominence as a viable alternative to conventional three-phase motor drives. These advantages encompass minimized torque pulsations, heightened power density, and enhanced capability for fault-tolerant [4, 5]. MPC is an increasingly prominent control method for drive systems, demonstrating superior performance and optimization [6]. Enhancing the performance of 5ph-IPMSM control plays a crucial role in advancing various innovative applications. In the drive systems, the tunning of PI controllers affects the speed response of the motor. The tuning is usually achieved by metahuristic techniques. Recently, RL are used instead to tune the PI controller [7] in DC drive system because it is an excellent multi-objective optimization technique due to its strong search capabilities and rapid convergence rate. To the best knowledge of the authors, no existing literature has investigated the optimization of the speed response of five-phase IPMSM based MPC using RL based TD3 algorithm across a wide speed range. Several authors presented several methods of control. These publications are explored as follows:

In [8], a harmonic elimination method utilizing vector control was introduced for the five-phase IPMSM. This method, known as harmonic elimination SVM, operates on the principles of space vector theory. In [9], a study was conducted on a 5ph-PMSM VC. A SVM algorithm was proposed to achieve high-performance VC in the drive system. Similarly, [10] introduced a VC strategy for a five-phase VSI utilizing SVM that powers a 5ph-PMSM. This approach optimized the performance of the 5ph-PMSM drive system. Furthermore, in [11], a mathematical model for the five-phase PMSM and corresponding control algorithms were presented. The modeling was achieved using coordinate transformation, and both the two-VC algorithm and four-VC algorithm SVPWMs were investigated. Additionally, a modified four-VC algorithm was introduced to modify the 3rd harmonic current, thereby improving the motor’s torque performance. In [12], a speed sensorless DTC method was proposed for five-phase IPMSM. This method relies on measuring the per-phase currents and the voltage of the DC bus. Additionally, [13] introduced a model reference adaptive control, incorporating a neural network, for a five-phase IPMSM. Combined with a hysteresis current controller, this controller enables motor speed control across a wide range of speeds. Furthermore, [14] presented a sensorless control methodology based on 3rd harmonic space for a five-phase PMSM, eliminating the need for motor parameters throughout the entire speed range. In [15], a super-twisting SMC was proposed to control a five-phase PMSM. This control technique exhibits superior response and performance compared to vector control in various conditions. DTMPC of a 5ph-PMSM drive system was presented in [16]. This control strategy effectively reduces the ripples of torque associated with conventional DTC, leading to reduced harmonics with high-orders and losses of the system. A PCC strategy based on a model of finite control set was introduced for a 5-ph PMSM in [17]. This approach reduces the THD to 9.47% and eliminates third harmonic currents. In [18], a MPDTC method was proposed based on the QEM method and a HVE method. The THD was reduced to 11.54% by employing this technique while disregarding third harmonic currents. [19] introduced a DTMPC technique for a five-phase PMSM. This approach aims to optimize torque development, improve speed regulation, reduce the ripples in torque, reduce current harmonics having higher orders, decrease losses in the drive system, and increase the power train’s efficiency. In [20], a MPTC technique based on double virtual voltage vectors utilizing geometric principles was presented for controlling a five-phase PMSM. This method achieves reduced processing time without the need for weighting factors. Additionally, [21] proposed an MPTC technique with additional weighting factors, reducing current harmonics and torque ripple. The THD was decreased to 7.11% using this method while disregarding third harmonic currents. Reference number [22] introduced a model-free PCC methodology. This methodology was based on a model called an ultra-local and the outputs of the motor for five-phase PMSM drives. This strategy mitigates the impact of motor parameter variations on current predictions. Moreover, [23] presented a MPCC methodology for a five-phase PMPSM, utilizing pre-selection for the voltage vector. The purpose of this methodology is to reduce computation time compared to conventional MPC by selecting the optimal voltage vector based on deviations in stator current and changes in the position of the stator current vector.

In [24], an ANN-based MPC strategy was suggested to control the speed of a 3-phase IPMSM. This approach utilized a predictive algorithm of back propagation (BP) network and MPC. The optimal selection of the gains of a PI controller in a VC three-phase PMSM drive system was addressed in [25]. Optimization algorithms called RGA and BBO were employed for this purpose. It was observed that using the BBO algorithm exhibited superior transient as well as steady-state performance in the PMSM drive system. In [26], a three-phase surface-mounted PMSM was controlled using an adaptive ANN internal model utilizing the PSO algorithm. Various optimization techniques were compared in [27] to obtain the optimal selection of PI controller gains for a three-phase PMSM drive system. A neural network-based MPC technique to reguate the speed of a 3-phase IPMSM was presented in [28]. Similarly, in [29], a control technique utilizing a BP ANN algorithm was given for a 3-phase IPMSM. Sensorless control of surface-mounted PMSM was achieved in [30] using an adaptive speed observer and a PID controller. In [31], a control technique for a three-phase IPMSM utilizing a MPC based on ANN was introduced. [32, 33] presented ANN-based MPC techniques for a three-phase IPMSM to overcome the effects of parameter mismatches. A dual-vector-based Particle Swarm Optimization MPC technique for a three-phase IPMSM was introduced in [34].

In [35] the reinforcement Q–learning algorithm was presented to tune fuzzy PD and PI controllers for SISO and TITO systems. The deep RL (DRL) was used to improve the tuning process for classical PID controller was presented in [36]. The algorithm that was used in RL is the DDPG. The concept of RL based FOC of a three-phase induction motor was presented in [37]. In [38] the design of adaptive RL PID controller under the structure of Actor-Critic which was based on RBF network for nonlinear systems was presented. Online training for a RL used to control real motor drive system was presented in [39]. The adopted drive system was composed of three-phase PMSM fed by VSI. In [40] DRL method for speed control of a three-phase PMSM servo drive system was presented. The presented DRL control improved the system performance especially in case of load variations. An adaptive PID controller using the algorithm of asynchronous advantage actor–critic was proposed in [41]. In [42] speed control of a PMSM is achieved by applying the PI based PSO and DDPG algorithms. In [43] a combined approach that leverages DRL and MPC was introduced to enhance the efficiency of electric vehicles. In [44] an open source toolbox called GEM was developed for training of RL-agents for the controlling of electric motors. The improved control performance for a three-phase PMSM was proposed in [45]. This improvement was achieved using four optimization techniques and RL. The used optimization techniques were PSO, SA, GA, and GWO. An RL control algorithm to control a three-phase PMSM based on the TD3 was suggested in [46]. In [47], an adaptive PID controller was designed for controlling the speed of a DC motor using RL based TD3 algorithm. In [48], a comparison was made between the conventional PID methodology and a TD3 RL algorithm to control three-phase PMSM based on the strategy of vector control (VC). In [7], researchers employed the TD3 method to learn PI controller for optimal dynamics in the simulation environment of controlling the speed of a DC motor. Energy management strategies for hybrid electric vehicles (HEVs) was dealt with in [49]. A hierarchical architecture that combines RL algorithms was proposed. The DDPG algorithm demonstrates superior performance in the energy management of HEVs. In [50], an intelligent system for energy management for a conventional autonomous vehicle using RL was proposed. A novel exploration strategy called self-adaptive Q-learning was introduced.

In this study, we suggest a novel RL control algorithm utilizing the TD3 approach. The algorithm is designed to fine-tune two cascaded PI controllers in a 5-phase voltage source inverter (VSI) / 5ph-IPMSM based MPC drive system. The primary goal of this control methodology is the optimization of the speed response of the 5ph-IPMSM under various operating conditions. We compare the speed responses obtained using the RL control algorithm with those achieved using four recent MHOT: TS, HBA, DM, and DO. The speed response are compared in terms of the settling time, rise time, maximum time and maximum overshoot percentage.

The following points summarize the main contributions of this paper:

- A new RL control algorithm utilizing the TD3 algorithm for tuninig the two cascaded PI controllers in the drive system under consideration is proposed.
- The tuning is achieved using the most recent optimization techniques: TS, HBA, DM, and DO.
- A comparative study is achieved among the suggested RL control methodology and the MHOT adopted in this research for the drive system under consideration.
- A MATLAB SIMULINK is accomplished for the drive system under consideration to obtain the results of simulation and verify the validity of the proposed control methodology.

The subsequent sections of this study are arranged as follows. In Section 2 the modeling of the drive system is introduced. Explanation of the MPC is given in Section 3. MHOT are explained briefly in Section 4. In Section 5, the suggested RL utilizing TD3 algorothm is explained. In Section 6, the proposed control methodology of the drive system is explored. In Section 7 simulation results are given. In Section 8, key conclusions drawn from our study are summarized.

## 2. Modeling of the drive system

### 2.1 Modeling of the five-phase VSI

The 5-phase VSI is expressed using the per-phase voltages (*v*_*a*_ to *v*_*e*_), which depend on the inverter switching functions [19]

$$\left[\begin{array}{c} v_{a}\\ v_{b}\\ v_{c}\\ v_{d}\\ v_{e} \end{array}\right]=\frac{V_{dc}}{5}\left[\begin{array}{ccccc} 4 & -1 & -1 & -1 & -1\\ -1 & 4 & -1 & -1 & -1\\ -1 & -1 & 4 & -1 & -1\\ -1 & -1 & -1 & 4 & -1\\ -1 & -1 & -1 & -1 & 4 \end{array}\right].\left[\begin{array}{c} s_{a}\\ s_{b}\\ s_{c}\\ s_{d}\\ s_{e} \end{array}\right]$$ (1)

In Eq (1), V_dc_ represents the DC voltage supplied to the inverter. The switching functions, denoted as S_a_ to S_e_, correspond to the various states of the inverter. Specifically, a switching function equals one when the upper semiconductor switch in a leg is active, and it equals zero when the lower switch is inactive in that leg.

In a five-phase VSI, there exist 32 possible switching states. The MPC determines the most suitable switching state to obtain minimum cost function, and based on these states, gate pulses are generated for the ten switches.

### 2.2 Model of the 5ph-IPMSM

The 5ph-IPMSM is described using the DQ model in the synchronous frame of reference. The voltage ABCDE to DQ transformations are provided by references [19, 51]:

$$\left[\begin{array}{c} v_{d1}\\ v_{q1}\\ v_{d3}\\ v_{q3}\\ v_{0} \end{array}\right]=\frac{2}{5}\left[\begin{array}{ccccc} \cos\theta & \cos(\theta -\alpha ) & \cos(\theta -2\alpha ) & \cos(\theta +2\alpha ) & \cos(\theta +\alpha )\\ -\sin\theta & -\sin(\theta -\alpha ) & -\sin(\theta -\alpha ) & -\sin(\theta +2\alpha ) & -\sin(\theta +\alpha )\\ \cos3\theta & \cos3(\theta -\alpha ) & \cos3(\theta -2\alpha ) & \cos3(\theta +2\alpha ) & \cos3(\theta +\alpha )\\ -\sin3\theta & -\sin3(\theta -\alpha ) & -\sin3(\theta -2\alpha ) & -\sin3(\theta +2\alpha ) & -\sin3(\theta +\alpha )\\ \frac{1}{\sqrt{2}} & \frac{1}{\sqrt{2}} & \frac{1}{\sqrt{2}} & \frac{1}{\sqrt{2}} & \frac{1}{\sqrt{2}} \end{array}\right].\left[\begin{array}{c} v_{a}\\ v_{b}\\ v_{c}\\ v_{d}\\ v_{e} \end{array}\right]$$ (2)

where α = 2π/5, θ is the rotor position angle, *v*_*d1*_, *v*_*q1*_ are the fundamental stator voltages in the DQ frame of reference, *v*_*d3*_, *v*_*q3*_ are the third-harmonic DQ components of stator voltage.

The transformation from the DQ frame of reference to the ABCDE frame of reference can be represented as follows [19]:

$$\left[\begin{array}{c} i_{as}\\ i_{bs}\\ i_{cs}\\ i_{ds}\\ i_{es} \end{array}\right]=\left[\begin{array}{ccccc} \cos\theta & -\sin\theta & \cos3\theta & -\sin3\theta & \frac{1}{\sqrt{2}}\\ \cos(\theta -\alpha ) & -\sin(\theta -\alpha ) & \cos3(\theta -\alpha ) & -\sin3(\theta -\alpha ) & \frac{1}{\sqrt{2}}\\ \cos(\theta -2\alpha ) & -\sin(\theta -2\alpha ) & \cos3(\theta -2\alpha ) & -\sin3(\theta -2\alpha ) & \frac{1}{\sqrt{2}}\\ \cos(\theta +2\alpha ) & -\sin(\theta +2\alpha ) & \cos3(\theta +2\alpha ) & -\sin3(\theta +2\alpha ) & \frac{1}{\sqrt{2}}\\ \cos(\theta +\alpha ) & -\sin(\theta +\alpha ) & \cos3(\theta +\alpha ) & -\sin3(\theta +\alpha ) & \frac{1}{\sqrt{2}} \end{array}\right].\left[\begin{array}{c} i_{d1}\\ i_{q1}\\ i_{d3}\\ i_{q3}\\ i_{0} \end{array}\right]$$ (3)

where *i*_*d1*_, *i*_*q1*_ are the fundamental stator currents DQ components, *i*_*d3*_, *i*_*q3*_ are the third-harmonic DQ components of stator currents. The DQ voltage equations for the 5ph-IPMSM, after removing the zero-sequence component, can be expressed as follows [19, 51]:

$$\left[\begin{array}{c} v_{d1}\\ v_{q1}\\ v_{d3}\\ v_{q3} \end{array}\right]=\left[\begin{array}{cccc} R_{s} & 0 & 0 & 0\\ 0 & R_{s} & 0 & 0\\ 0 & 0 & R_{s} & 0\\ 0 & 0 & 0 & R_{s} \end{array}\right].\left[\begin{array}{c} i_{d1}\\ i_{q1}\\ i_{d3}\\ i_{q3} \end{array}\right]+.\left[\begin{array}{c} \frac{d\lambda_{d1}}{dt}\\ \frac{d\lambda_{q1}}{dt}\\ \frac{d\lambda_{d3}}{dt}\\ \frac{d\lambda_{q3}}{dt} \end{array}\right]-\omega \left[\begin{array}{cccc} 0 & 1 & 0 & 0\\ -1 & 0 & 0 & 0\\ 0 & 0 & 0 & 3\\ 0 & 0 & -3 & 0 \end{array}\right].\left[\begin{array}{c} \lambda_{d1}\\ \lambda_{q1}\\ \lambda_{d3}\\ \lambda_{q3} \end{array}\right]$$ (4)

where *R*_*s*_ is the stator resistance, ω is the motor speed in electrical rad/sec, *λ*_*d1*_, *λ*_*q1*_ are the fundamental DQ stator flux linkages and *λ*_*d3*_, *λ*_*q3*_ are the 3^rd^ harmonic DQ stator flux linkages. The DQ stator flux linkages take the following form:

$$\begin{array}{l} \lambda_{d1}=L_{d1}i_{d1}+L_{m13}i_{d3}+\lambda_{1m}\\ \lambda_{q1}=L_{q1}i_{q1}+L_{m13}i_{q3}\\ \lambda_{d3}=L_{d3}i_{d3}+L_{m13}i_{d1}+\lambda_{3m}\\ \lambda_{q3}=L_{q3}i_{q3}+L_{m13}i_{q1} \end{array}$$ (5)

where *L*_*d1*_, *L*_*q1*_ are the fundamental direct and quadrature self-inductances, *L*_*m13*_ is the mutual inductance and *λ*_*1m*_ and *λ*_*3m*_ are the fundamental and 3^rd^ harmonic components of the rotor PM flux linkages respectively.

The 5ph-IPMSM differential equation, Eq (4) can be rearranged to take the following formula:

D[I]=[LL][V]−[LR].[I]−ω[Lλ].[I]+ω[LG].[I] (6)

where D is the operator d/dt, $[I]={\left[\begin{array}{cccc} i_{d1} & i_{q1} & i_{d3} & i_{q3} \end{array}\right]}^{T}$, $[V]={\left[\begin{array}{cccc} v_{d1} & v_{q1} & v_{d3} & v_{q3} \end{array}\right]}^{T}$,

$$[LL]=\left[\begin{array}{cccc} \frac{-L_{d3}}{L_{m13}^{2}-L_{d1}L_{d3}} & 0 & \frac{L_{m13}}{L_{m13}^{2}-L_{d1}L_{d3}} & 0\\ 0 & \frac{-L_{q3}}{L_{m13}^{2}-L_{q1}L_{q3}} & 0 & \frac{L_{m13}}{L_{m13}^{2}-L_{q1}L_{q3}}\\ \frac{L_{m13}}{L_{m13}^{2}-L_{d1}L_{d3}} & 0 & \frac{-L_{d1}}{L_{m13}^{2}-L_{d1}L_{d3}} & 0\\ 0 & \frac{L_{m13}}{L_{m13}^{2}-L_{q1}L_{q3}} & 0 & \frac{-L_{q1}}{L_{m13}^{2}-L_{q1}L_{q3}} \end{array}\right]$$

$$[LR]=\left[\begin{array}{cccc} \frac{-L_{d3}R_{s}}{L_{m13}^{2}-L_{d1}L_{d3}} & 0 & \frac{L_{m13}R_{s}}{L_{m13}^{2}-L_{d1}L_{d3}} & 0\\ 0 & \frac{-L_{q3}R_{s}}{L_{m13}^{2}-L_{q1}L_{q3}} & 0 & \frac{L_{m13}R_{s}}{L_{m13}^{2}-L_{q1}L_{q3}}\\ \frac{L_{m13}R_{s}}{L_{m13}^{2}-L_{d1}L_{d3}} & 0 & \frac{-L_{d1}R_{s}}{L_{m13}^{2}-L_{d1}L_{d3}} & 0\\ 0 & \frac{L_{m13}R_{s}}{L_{m13}^{2}-L_{q1}L_{q3}} & 0 & \frac{-L_{q1}R_{s}}{L_{m13}^{2}-L_{q1}L_{q3}} \end{array}\right]$$

$$[LG]=\left[\begin{array}{cccc} 0 & \frac{3L_{m13}^{2}-L_{d3}L_{q1}}{L_{m13}^{2}-L_{d1}L_{d3}} & 0 & \frac{3L_{m13}L_{q3}-L_{d3}L_{m13}}{L_{m13}^{2}-L_{d1}L_{d3}}\\ \frac{L_{d1}L_{q3}-3L_{m13}^{2}}{L_{m13}^{2}-L_{q1}L_{q3}} & 0 & \frac{L_{m13}L_{q3}-3L_{d3}L_{m13}}{L_{m13}^{2}-L_{q1}L_{q3}} & 0\\ 0 & \frac{L_{m13}L_{q1}-3L_{d1}L_{m13}}{L_{m13}^{2}-L_{d1}L_{d3}} & 0 & \frac{L_{m13}^{2}-3L_{d1}L_{q3}}{L_{m13}^{2}-L_{d1}L_{d3}}\\ \frac{3L_{m13}L_{q1}-L_{d1}L_{m13}}{L_{m13}^{2}-L_{q1}L_{q3}} & 0 & \frac{3L_{d3}L_{q1}-L_{m13}^{2}}{L_{m13}^{2}-L_{q1}L_{q3}} & 0 \end{array}\right]$$

$$[L\lambda ]=\left[\begin{array}{c} 0\\ \frac{3\lambda_{3m}L_{m13}-\lambda_{1m}L_{q3}}{L_{m13}^{2}-L_{q1}L_{q3}}\\ 0\\ \frac{\lambda_{1m}L_{m13}-3\lambda_{3m}L_{q1}}{L_{m13}^{2}-L_{q1}L_{q3}} \end{array}\right]$$

The motor torque equation can be represented as [19, 51]:

$$T_{e}=\frac{5}{2}\frac{p}{2}[\lambda_{d1}i_{q1}-\lambda_{q1}i_{d1}+3\lambda_{d3}i_{q3}-3\lambda_{q3}i_{d3}]$$ (7)

Substitution of Eq (5) into Eq (7) gives [19]:

$$T_{e}=\frac{5}{2}\frac{p}{2}[(L_{d1}-L_{q1})i_{d1}i_{q1}+2L_{m13}(i_{d1}i_{q3}-i_{q1}i_{d3})+3(L_{d3}-L_{q3})i_{d3}i_{q3}+(\lambda_{1m}i_{q1}+3\lambda_{3m}i_{q3})]$$ (8)

where p is the poles total number. When considering motor speed variations during the transient time interval, Eq (6) becomes nonlinear. As a result, the 5ph-IPMSM currents need to be numerically solved. To achieve this, we employ the 5ph-IPMSM mechanical equation, which is expressed as:

$$D\omega_{m}=\frac{T_{e}-T_{l}(\omega_{m})}{J}$$ (9)

In the given equation, the motor speed is denoted by *ω*_*m*_ in mechanical radians per second, J denotes the inertia, and *T*_*l*_(*ω*_*m*_) is defined as follows:

$$T_{l}(\omega_{m})=T_{L}+T_{fw}$$ (10)

Where *T*_*L*_ and *T*_*fw*_ are the load and friction and windage torques respectively.

### 2.3 Five-phase IPMSM maximum torque per ampere operating mode of operation model

To maximize efficiency, the 5ph-IPMSM operates at maximum torque per ampere, particularly for speeds up to the motor’s rated speed. This section derives the reference fundamental and 3^rd^ harmonic DQ currents components.

When L_m13_ is disregarded, the torque equation for the fundamental component can be formulated using Eq (8) as follows:

$$T_{e1}=\frac{5}{2}\frac{p}{2}[(L_{d1}-L_{q1})i_{d1}i_{q1}+\lambda_{1m}i_{q1}]$$ (11)

The reference fundamental direct current component is derived by differentiating the fundamental torque equation, Eq (11), w.r.t. the fundamental direct current and setting the result to zero, i.e. $\frac{dT_{e1}}{di_{d1}}=0$. Consequently, the reference fundamental direct current component is determined as shown in the following equation:

$$i_{d1}=\frac{\lambda_{1m}}{2(L_{q1}-L_{d1})}-\sqrt{\frac{\lambda_{1m}^{2}}{4{\left(L_{q1}-L_{d1}\right)}^{2}}+i_{q1}^{2}}$$ (12)

The reference fundamental quadrature current component can be derived from Eq (11) as follows:

$$i_{q1}=\frac{T_{e1}}{\frac{5}{2}\frac{p}{2}[(L_{d1}-L_{q1})i_{d1}+\lambda_{1m}]}$$ (13)

The reference 3^rd^ harmonic direct current component can be obtained from [52]:

$$i_{d3}=k\sqrt{i_{d1}^{2}+i_{q1}^{2}}\sin\{3[\tan^{-1}(\frac{i_{d1}}{i_{q1}})]\}$$ (14)

Also, the reference 3^rd^ harmonic quadrature current component can be obtained from [52]:

$$i_{q3}=k\sqrt{i_{d1}^{2}+i_{q1}^{2}}\cos\{3[\tan^{-1}(\frac{i_{d1}}{i_{q1}})]\}$$ (15)

### 2.4 Five-phase IPMSM field weakening operating mode model

To extend the operating speed range beyond the rated speed, the 5ph-IPMSM will be operated in field weakening mode. The equations for the reference fundamental and 3^rd^ harmonic DQ currents components are derived as follows.

The fundamental direct and quadrature steady-state voltages can be obtained from Eq (4) and neglecting the stator resistance voltage drops, we have:

The fundamental direct and quadrature steady-state voltages can be derived from Eq (4). By neglecting the stator resistance voltage drops, we obtain the following equations:

$$\begin{array}{l} v_{d1}=-\omega \lambda_{q1}\\ v_{q1}=\omega \lambda_{d1} \end{array}$$ (16)

By substituting *λ*_*d*1_ and *λ*_*q*1_ from Eq (5) into Eq (16) and ignoring L_m13_, we obtain:

$$\begin{array}{l} v_{d1}=-\omega L_{q}i_{q1}\\ v_{q1}=\omega L_{d}i_{d1} \end{array}$$ (17)

The reference fundamental direct and quadrature components should satisfy the following equation to guarantee the maximum fundamental voltage, V_m1_, of the IPMSM:

The reference fundamental direct and quadrature components must satisfy the following equation to ensure the maximum fundamental voltage, V_m1_, of the 5ph-IPMSM:

$$V_{m1}=\sqrt{v_{d1}^{2}+v_{q1}^{2}}$$ (18)

By substituting Eq (17) into Eq (18) and solving for the fundamental direct current component, we obtain:

$$i_{d1}=\frac{-\lambda_{1m}}{L_{d1}}+\frac{1}{L_{d1}}\sqrt{\frac{V_{1m}^{2}}{\omega^{2}}-L_{q1}^{2}i_{q1}^{2}}$$ (19)

In this mode of operation, equations analogous to Eqs. (13), (14), and (15), expressed in terms of the fundamental direct current component provided in Eq (19), are utilized.

## 3. Model predictive control technique

The MPC consists of two primary components: the modelof the plant and the optimizer. The core concept of MPC is to choose the optimal sequence of inputs for the plant utilizing predictions of its future behavior. These predictions are achieved using the modelof the plant, which employs previous states to predict future states. At each discrete interval, the optimizer leverages the predicted states and the desired trajectory to address the optimization problem over the prediction horizon, thereby identifying the optimal set of inputs for future operations.

To successfully implement MPC, it is crucial to discretize the motor model. Thus, Eq (6) is converted into its discrete form using the Forward Euler approximation method. Consequently, the discrete model for the 5-phase IPMSM can be represented as follows:

$$\left[I(k+1)]=[I(k)]+T_{s}\{[LL][V]-[LR].[I(k)]-\omega [L\lambda ].[I(k)]+\omega [LG].[I(k)]\right\}$$ (20)

In this context, T_s_ denotes the sampling interval of the discretized system.

The MPC is specifically designed to minimize torque error as its primary objective. Since the electromagnetic torque equation for a five-phase IPMSM involves contributions from both the d-q axes currents, it is necessary to control these currents to reduce the error in torque. Consequently, the problem can be simplified by minimizing the error in current error instead of torque error. Therefore, the basic CF aimed at reducing current error can be formulated as follows:

The primary objective of the MPC is to reduce torque error. Given that the electromagnetic torque equation for a 5ph-IPMSM includes contributions from both the d-q axes currents, controlling these currents is essential to decrease torque error. Thus, the problem can be made simpler by focusing on reducing current error instead of torque error. Therefore, the basic cost function (CF) aimed at reducing current error can be formulated as follows:

$$C.F={\left[i_{d1r}-i_{d1}(k+1)\right]}^{2}+{\left[i_{q1r}-i_{q1}(k+1)\right]}^{2}+{\left[i_{d3r}-i_{d3}(k+1)\right]}^{2}+{\left[i_{q3r}-i_{q3}(k+1)\right]}^{2}$$ (21)

Where *i*_*d1r*_, *i*_*q1r*_, *i*_*d3r*_, *i*_*q3r*_ represent the reference direct and quadrature fundamental and third harmonic currents. These currents are determined using either Eqs. (12), (13), (14), and (15) for motor operation in the region of constant torque, or Eqs. (19), (13), (14), and (15) motor operatin in the region of constant power. The optimal inverter switching functions are chosen based on the minimum cost function.

## 4. Metahuristic optimization techniques

### 4.1 PI controller

The PI (proportional-integral) controller is commonly utilized in various industrial applications because of its simplicity, ease of implementation, and robust performance. Achieving optimal performance with a PI controller involves fine-tuning two key parameters: the proportional gain (*k*_*p*_) and the integral gain (*k*_*i*_). To optimize the performance of PI controllers, researchers have employed various optimization algorithms. In this study, four of the most recent optimization techniques are used for obtaining the optimum PI gains. These techniques are Transit Search (TS) [53], Honey Badger Algorithm (HBA) [54], Dwarf Mongoose (DM) [55], and Dandelion-Optimizer (DO) [56]. These techniques are compared with the RL utilizing TD3 algorithm. The goal is to minimize the error associated with the control of speed of a 5-phase IPMSM, to ensure the best possible performance.

The optimization process involves reduccing the error e(t), which is generated by comparing the reference speed with the actual speed, as well as the reference torque and actual torque, using four standard performance indicators: IAE, ISE, ITAE, and ITSE. This optimization is achieved through the utilization of the following equation, Eq (22), that accurately represents the superior results obtained with the proposed control technique.


$$\left\{ \begin{array}{c} IAE=\int_{0}^{tss}|e(t)|\cdot dt\\ ISE=\int_{0}^{tss}e^{2}(t)\cdot dt\\ ITAE=\int_{0}^{tss}t.|e(t)|\cdot dt\\ ITSE=\int_{0}^{tss}t.e^{2}(t)\cdot dt \end{array}\right.$$ (22)

The optimization objective involves minimizing the steady-state time response (*tss*) and the error function e(t).

### 4.2 Objective

The optimization problem involves the simultaneous pursuit of two distinct objectives, allowing for a comprehensive optimization strategy tailored to address the specific aims of each target. The optimization focuses on minimizing the speed and torque errors of the individual controllers by fine-tuning their respective gain parameters.

There are two PI controllers—one for the regulation of speed and another for controlling the torque. Each controller has two adjustable gains, the proportional and integerals gains (*k*_*p*_ and *k*_*i*_). Optimization must determine the optimal values for these four gain parameters, which are constrained within the range of [0, 300] for each gain. By optimizing these controller gains, the goal is to achieve high precision in both speed and torque regulation, thereby enhancing the overall system performance. The optimization strategy must navigate this multi-objective landscape to identify the set of gain values that best satisfies the conflicting targets of minimizing both speed and torque errors simultaneously.


Target=min(error)

$$error=\int {\left(T_{act}-T_{ref}\right)}^{2}dt+\int {\left(w_{act}-w_{ref}\right)}^{2}dt$$ (23)

## 5. Reinforcement learning based TD3 approach

The TD3 approach is an advancement over the DDPG algorithm. TD3 addresses the function approximation error that can occur in DDPG. The TD3 algorithm significantly enhances both the learning speed and performance of DDPG across various challenging continuous control tasks. The algorithm of TD3 outperforms many state-of-the-art methods. Due to the simplicity of TD3 modifications, they can be easily integrated into any other actor-critic algorithm [57, 58]. In addition to this, the TD3 algorithm learns two Q-value functions and uses the minimum estimate during policy updates.

It does this by using twin critic networks (CN), delayed target network updates, and added exploration noise, which together help to stabilize the process of training and improve the efficiency of the learned policy. The goal of RL is to obtain the optimum policy *π* that makes the predectid rewared to have maximum value which is achieved by tuning the parameter. This is typically achieved by renewing the parameter by employing the gradient ∇_*φ*_*J*(*φ*) RL employs an actor-critic structure, where the policy (actor) is renewed according to the DPG algorithm. In case of large value of state space, the Q-value function *Q*(*s*, *a*) is nearly determined using a function approximator *Q*_*θ*_’(*s*, *a*) with the aiding of a tuning parameter *θ*. To maintain a stable learning objective, a frozen target network *Q*_*θ*_’(*s*, *a*) is used to maintain a stable learning goal y across many updates [57].


$$y=r+\gamma Q_{\theta^{\prime }}\left(s^{\prime },a^{\prime }\right),\;a^{\prime }\leftarrow \pi_{\varphi^{\prime }}\left(s^{\prime }\right)$$ (24)

Actions are selected by the algorithm from a desired actor-network (AN) *π*_*φ*_^’^(*s*) that is separated from the main AN. The weights of the target network are periodically renewed to accurately approximate the weights of the current network using a soft update rule [57]. This helps maintain a stable learning objective during the training process.

In actor-critic (A-C) methods, the current and desired networks may be very similar, leading to inaccurate value prediction. I order to address this, the algorithm uses a set of two actors $\left(\pi_{\varphi_{1}},\pi_{\varphi_{2}}\right)$ and critics $\left(Q_{\theta_{1}},Q_{\theta_{2}}\right)$ [57]. The actors are optimized according to their respective critics, but this can cause overestimation. To mitigate this, a trimmed double Q-learning approach is used, that selects the minimum of the two critic estimates as the target update [58]. The AN and CN are then updated according to this formulation.


$$y=r+\underset{i=1,2}{\min}Q_{\theta^{\prime }}\left(s^{\prime },\pi_{\varphi_{1}}\left(s^{\prime }\right)\right)$$ (25)

The algorithm uses a reward value *r* and a discount factor *γ* which determines the influence of previous reward values on next decisions. The value of discount factor *γ* ranges from 0 to 1 [57, 59]. To address the issue of deterministic policies overfitting sharp peaks in the value approximation, the algorithm adds a tiny amount of random noise to the desired policy, which is then trimmed to keep the objective within a limited range [57].


$$y=r+\underset{i=1,2}{\min}Q_{\theta_{i}^{\prime }}\left(s^{\prime },\pi_{\varphi_{1}}\left(s^{\prime }\right)+\in \right)\;\in \approx \mathrm{clip}\;(N(0,\sigma ),-c,c)$$ (26)

**Fig 1:**
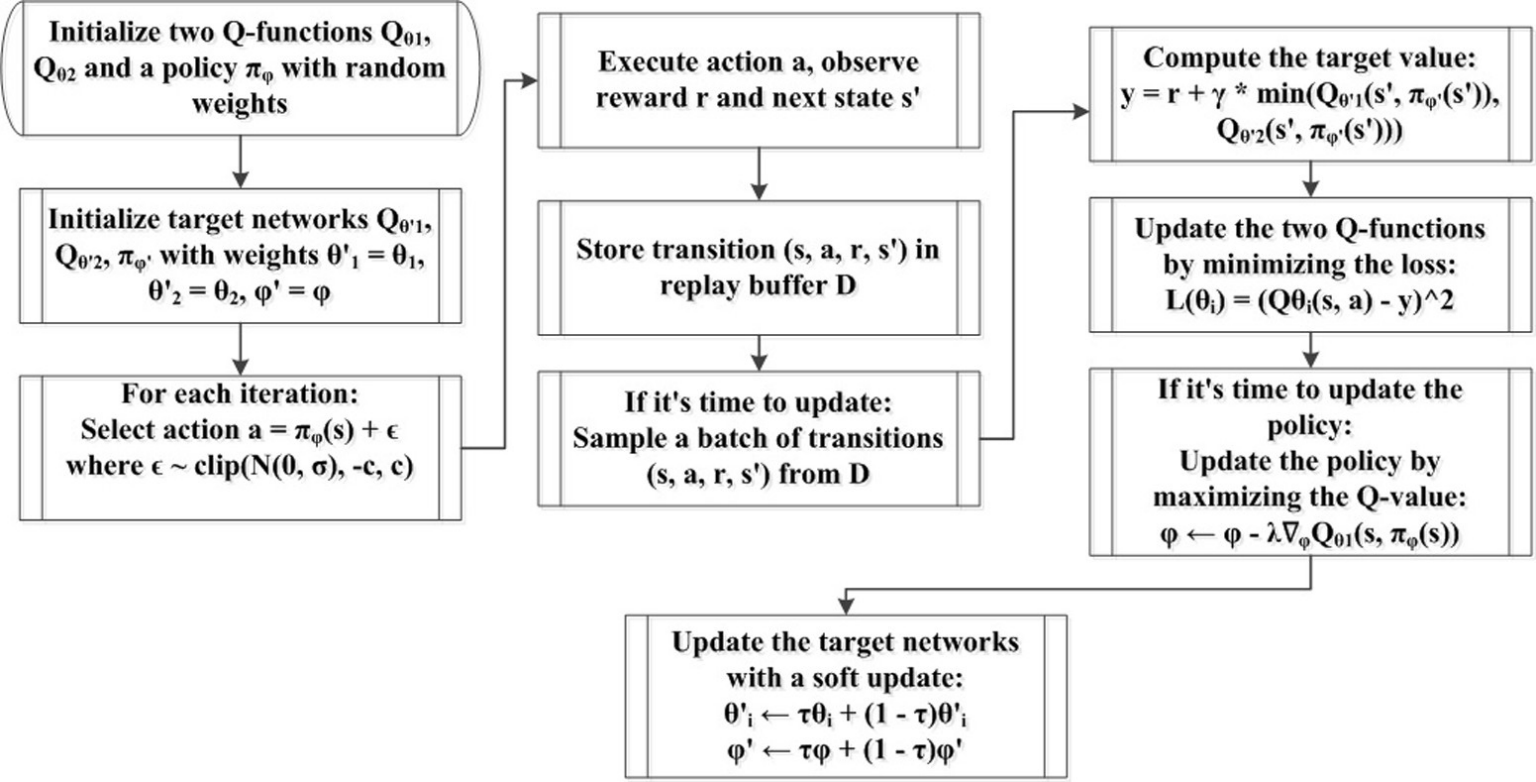
TD3 algorithm.

The TD3 approach is presented as flowchart in Fig 1, as proposed by [57, 60]. Table 1 lists some of the key parameters used in the TD3 algorithm.

**Table 1:** Parameters for TD3 approach [60].

Parameters	Explanations
$Q_{\theta_{1}},Q_{\theta_{2}}$	two CNs, where *θ*_1_ and *θ*_2_ represent the respective weights of the CNs.
$Q_{\theta_{1}^{\prime }},Q_{\theta_{2}^{\prime }}$	two target CNs, where $\theta_{1}^{\prime }$ and $\theta_{2}^{\prime }$ represent the respective weights of the target CNs.
*π*_*φ*_,*π*_*φ*^′^_	AN, *π*_*φ*_, and a target AN, *π*_*φ*^′^_, where *φ* represents the weights of the AN and *φ*^′^ represents the weights of the target AN.
tuple (s,a,r,s^′^)	The system transition tuples, where *s* represents the current state, *a* is the action taken, *r* is the reward received, and *s*^’^ is the next state.
*γ*	A CN discount factor
$\theta_{1}^{\prime },\theta_{2}^{\prime }$	The weight parameters are updated according to the next value calculation.
*φ* ^′^	represent the next value for the AN.
*τ*	represent the weights used for Polyak averaging.

**Fig 2:**
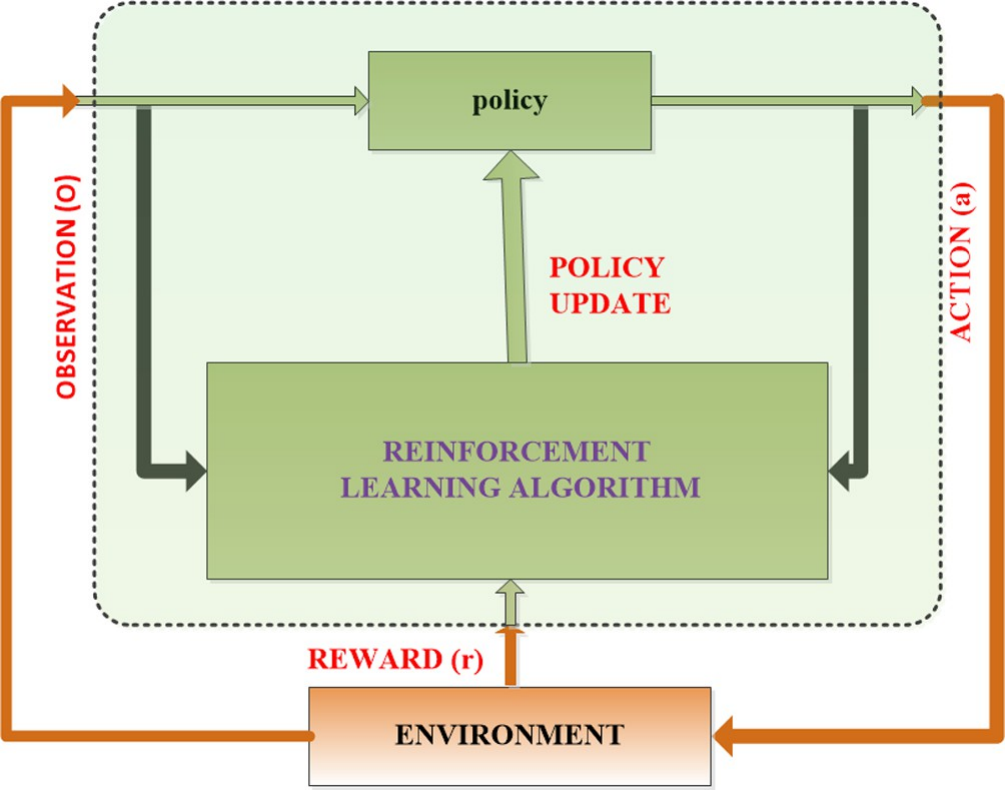
RL block diagram.

The block diagram depicted in Fig 2 illustrates the RL agent utilizing the TD3 algorithm for process control. In this context, the goal of obtaining the optimum policy for the RL-TD3 agent may be interpreted as determining the appropriate command signals for the 5ph-PMSM in order to ehance a given effectiveness metric.

The simulation environment of Matlab Simulink is utilized to implement the TD3 algorithm. The objective is to train an optimum two-stage PI controller for regulating the speed and torque of a 5ph-IPMSM in the control system setup. The process of learning generates the optimum tuning parameters for the two PI controllers, which can effectively address the regulation challenges simulated in the system environment.

**Fig 3:**
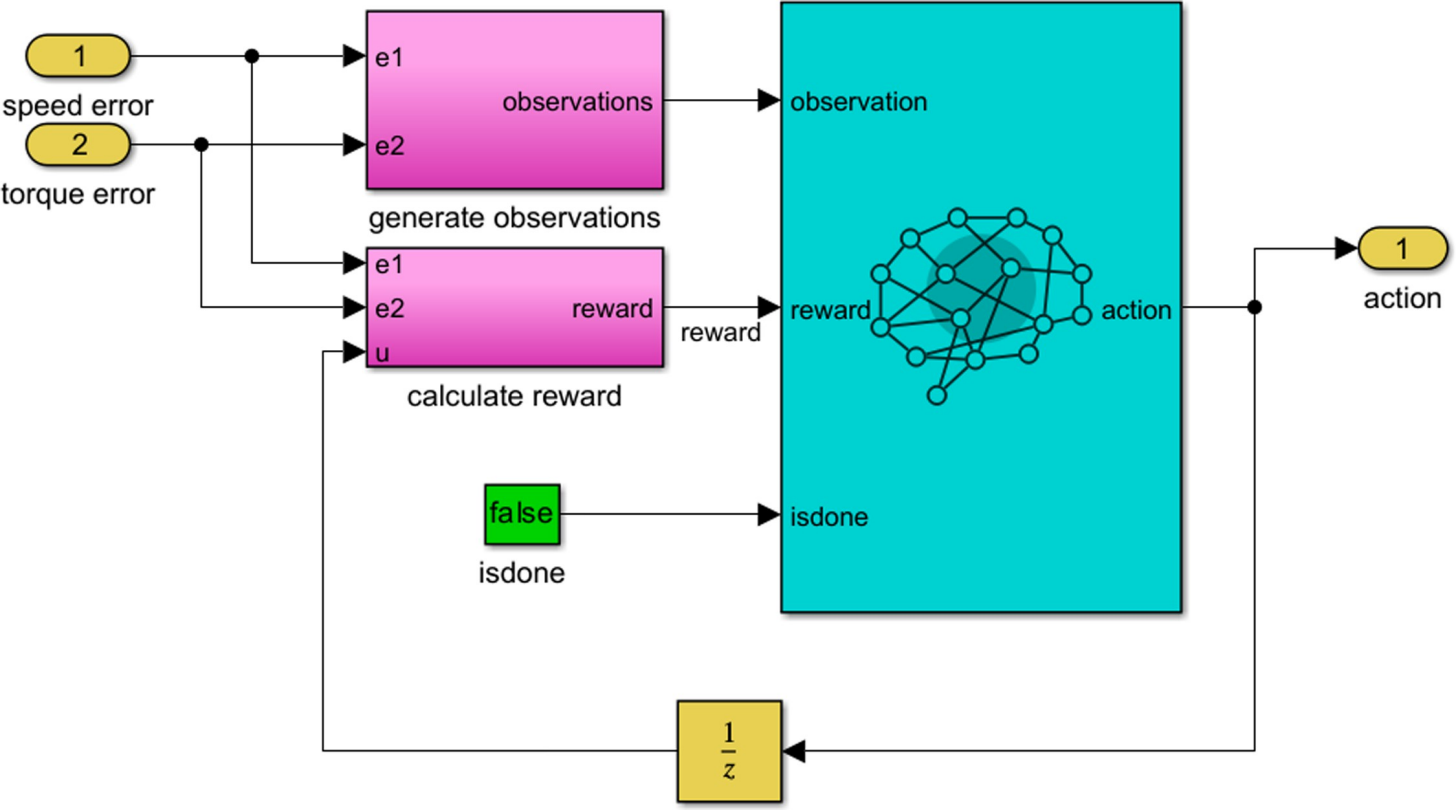
Matlabsimulink block diagram for the TD3 approach.

This setup can be viewed as analogous to the control of an industrial process, where the observed inputs are the speed error and its integral, as well as the torque, as shown in Fig 3 while the outputs represent the reference signals, and the enhancement objective is framed as a reward function.

The reward function is defined by the subsequent expression:

$$r(t)=-\left(w_{c1}\int_{0}^{t}e_{1}{\left(t\right)}^{2}dt+w_{c2}\int_{0}^{t}e_{2}{\left(t\right)}^{2}dt+w_{c3}u{\left(t\right)}^{2}\right)$$ (27)

The weight coefficients *w*_*c*1_,*w*_*c*2_, and *w*_*c*3_ are used to balance the reduction of the reference error and the control signal value. The error signals *e*_1_(*t*),*e*_2_(*t*) represent the observed states from the environment of the control system, and the control signal u(t) corresponds to the action of the actor.

The optimal control reward equation can be represented by:

$$R(z)=-W_{c1}\frac{T_{s}}{z-1}e_{1}{\left(z\right)}^{2}-W_{c2}\frac{T_{s}}{z-1}e_{2}{\left(z\right)}^{2}-W_{c3}u{\left(t\right)}^{2}$$ (28)

**Fig 4:**
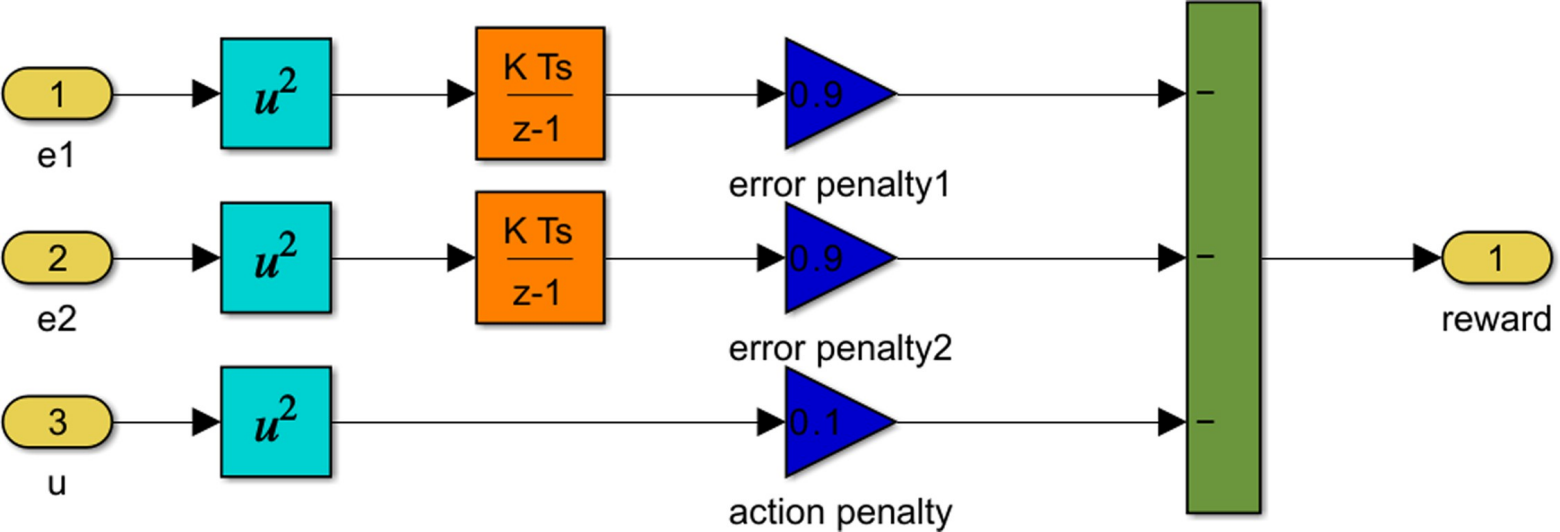
Matlab Simulink block diagram of the reward equation.

Fig 4 shows the Matlab Simulink block diagram of reward equation, Eq (28). In this figure the weights, *W*_*c*1_,*W*_*c*2_ and *W*_*c*3_ are taken to be 0.9, 0.9 and 0.1 respectively [7].

**Fig 5:**
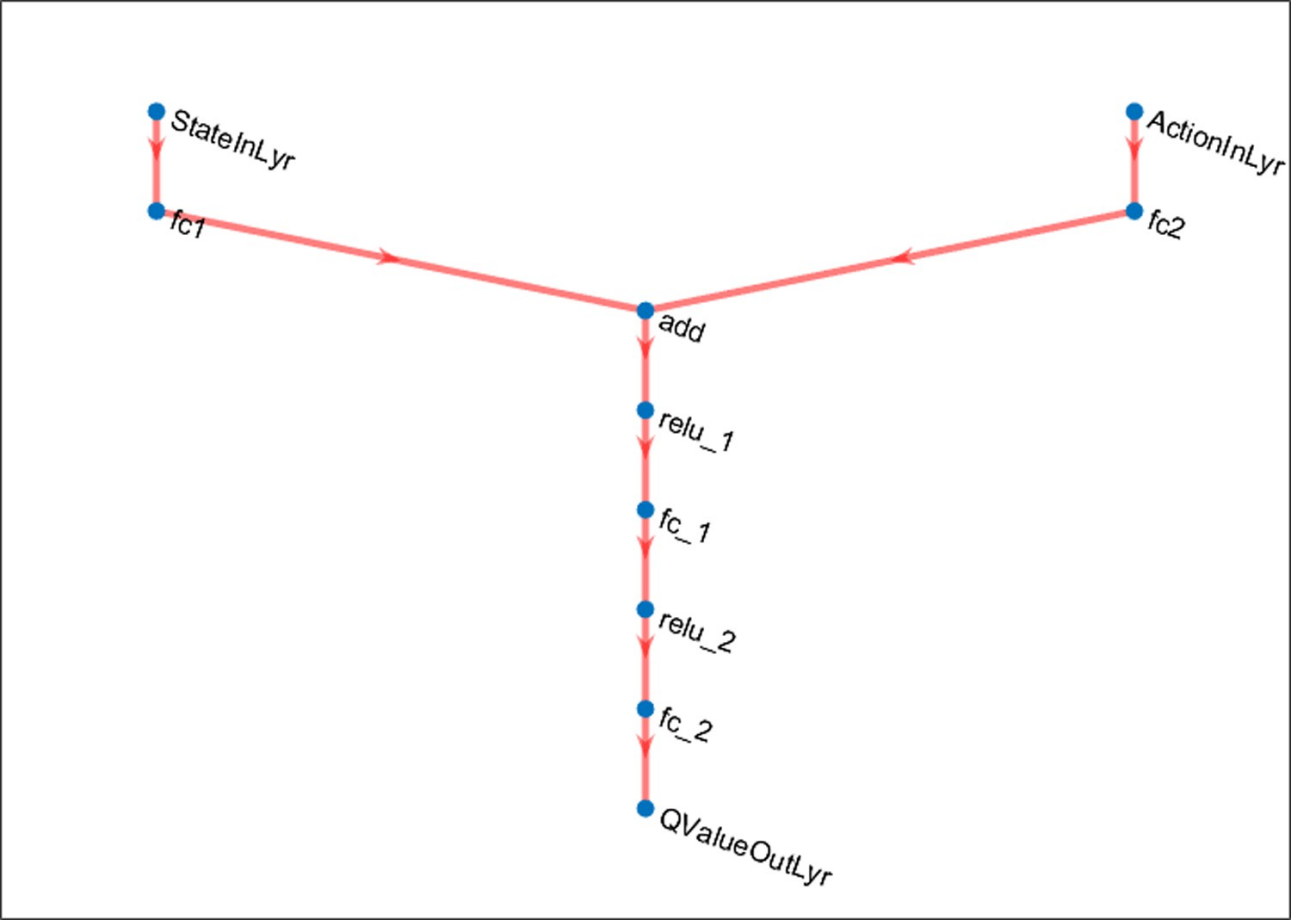
The architecture of CNs utilized in the drive system under consideration.

The AN and CN, with their corresponding reference networks, were set up in the MATLAB environment to achieve RL agents using the TD3 approach. The CN utilize the action (*a*) and observed state (*s*) as inputs, and function as an approximation to approximate the quality value *Q*(*s*, *a*). The arrangement of CNs utilized in the drive system under consideration is given in Fig 5.

The CNs consist of three layers:

- fully connected (fc) input layers for the action and state inputs,
- a fc common path layer,
- a fc output layer with one neuron representing Q(s, a).

The input and common path layers use ReLU activation. The actor networks take the observed state (*s*) as input and output is the action (*a*). The TD3 algorithm trains the AN and CN to enhance the reward, which is the optimum control goal [61].

The gains of the PI controllers k_i1_, k_P1_, k_i2_ and k_p2_ can be found using the Matlab functions: *actor = getActor (agent)* and *parameters = getLearnableParameters (actor)*.

## 6. Proposed control methodology

**Fig 6:**
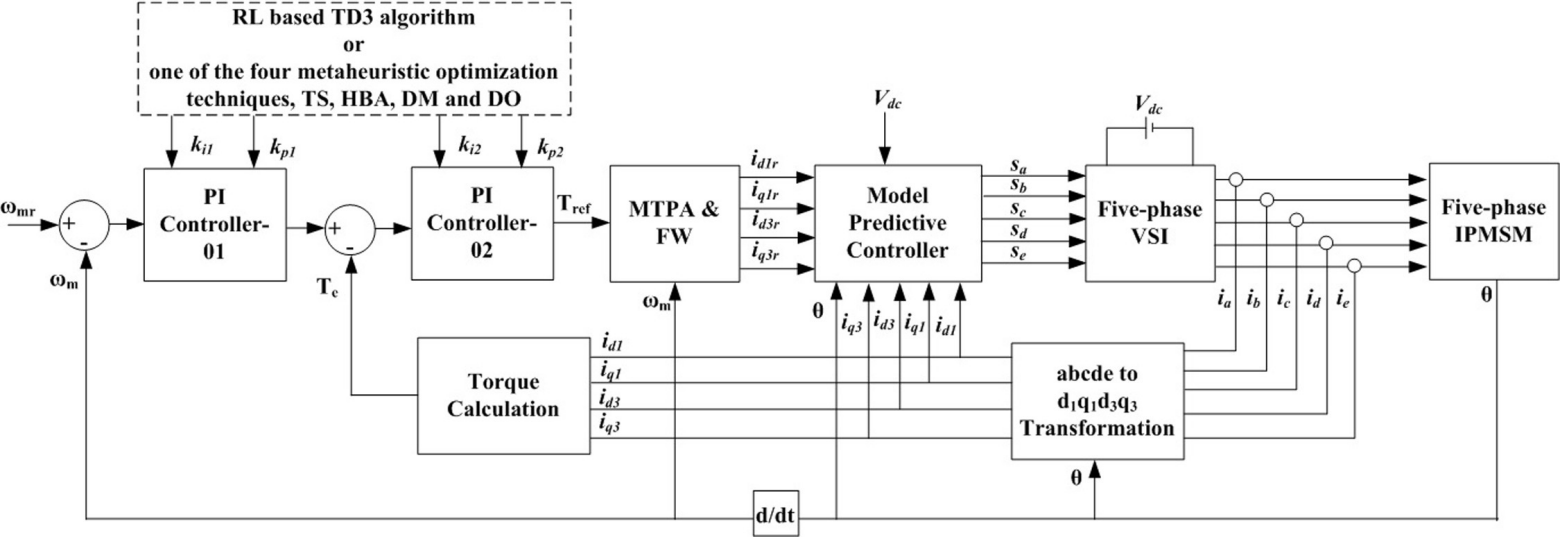
Proposed 5ph-IPMSM control system.

Fig 6 illustrates the proposed control system, which comprises several blocks. The rotor position is measured at a specific load torque and reference speed of the 5ph-IPMSM to determine the actual motor speed, as shown in Fig 6. The speed error is processed by a primary PI controller to generate the reference torque. The motor currents of the 5-ph IPMSM are measured and transformed into the DQ frame of reference using the “abcde to d1q1d3q3 Transformation” block in Fig 6. This block represents Eq (2). The resulting fundamental and 3^rd^ direct and quadrature currents are used to calculate the electromagnetic torque via the “Torque Calculation” block, which corresponds to Eq (8).

The gains of the primary and secondary PI controllers (k_i1_, k_P1_, k_i2_, and k_p2_) are determined using one of four metaheuristic optimization techniques (TS, HBA, DM, and DO) or the presented RL-based TD3 algorithm. The torque error is processed by the secondary PI controller, “PI Controller-02,” to obtain the corrected reference torque, T_ref_. The motor speed, ω_m_, and T_ref_ are used to derive the reference DQ currents from the “MTPA & FW” block in Fig 6. This block represents Eqs (12), (13), (14), and (15) for MTPA operation when the motor speed is not greater than the rated speed, and Eqs (19), (13), (14), and (15) for FW operation when the motor speed exceeds the rated speed.

The reference fundamental and 3^rd^ harmonic DQ currents, along with the rotor position angle and DC voltage, are used in the MPC, Eq (20), to generate the inverter gating signals that satisfy the cost function given in Eq (21) to minimize torque ripples and thus minimizing ripples in the motor speed.

## 7. Results

Several sets of results are obtained to prove the correctness of the suggested control methodology for the 5ph-IPMSM. The 5ph-IPMSM parameters are given in Table 2 [35].

**Table 2:** Five-phase IPMSM parameters.

Parameter	Value
poles (p)	4 poles
Rated power	12 kW
Rated Speed	1800 rpm
Maximum speed	5400 rpm
Rated Torque	63.662 Nm
*R*_*s*_	0.389 Ω
*L*_*d1*_	2.7 mH
*L*_*q1*_	9.6 mH
*L*_*d3*_	1.1 mH
*L*_*q3*_	2 mH
*L*_*m13*_	0 mH
*λ*_*1m*_	0.11 WbT
*λ*_*3m*_	0.0012 WbT
Moment of inertia	0.0036 kg.m^2^
Connection	Star

**Fig 7:**
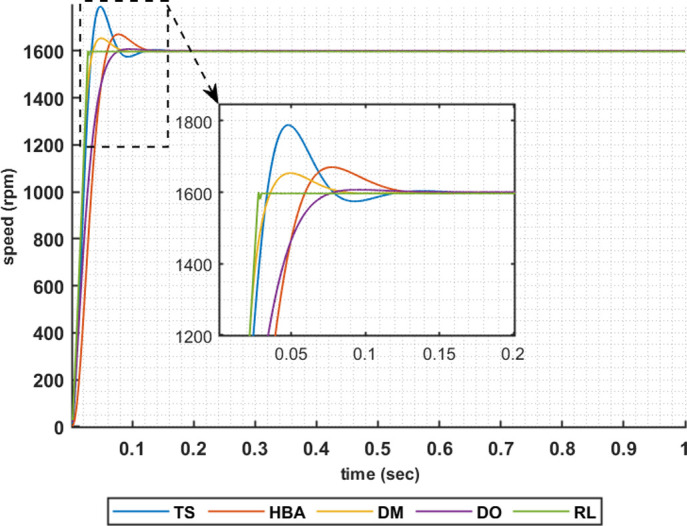
The motor speed responces when IAE error criteria is used.

**Fig 8:**
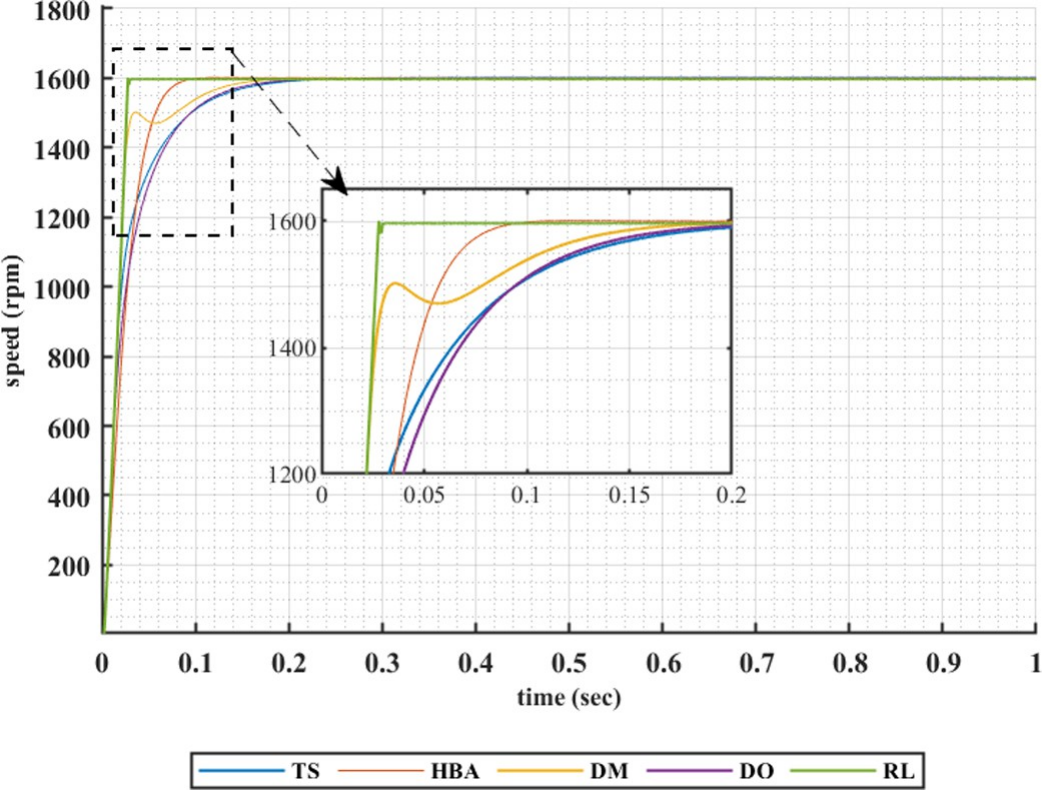
The motor speed responces when ISE error criteria is used.

**Fig 9:**
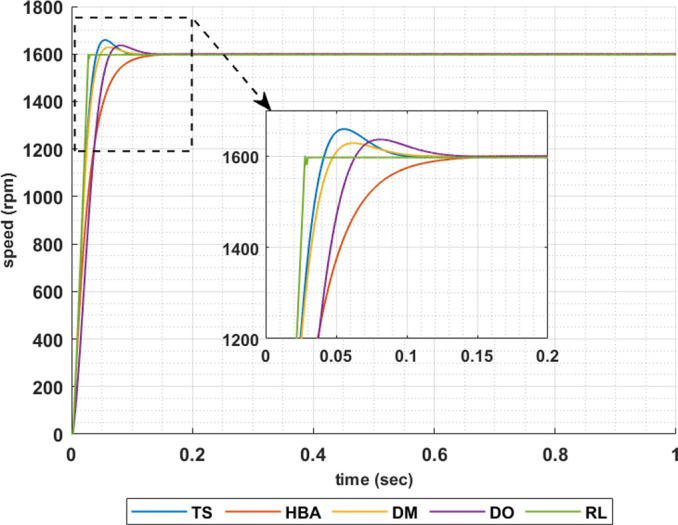
The motor speed responces when ITAE error criteria is used.

**Fig 10:**
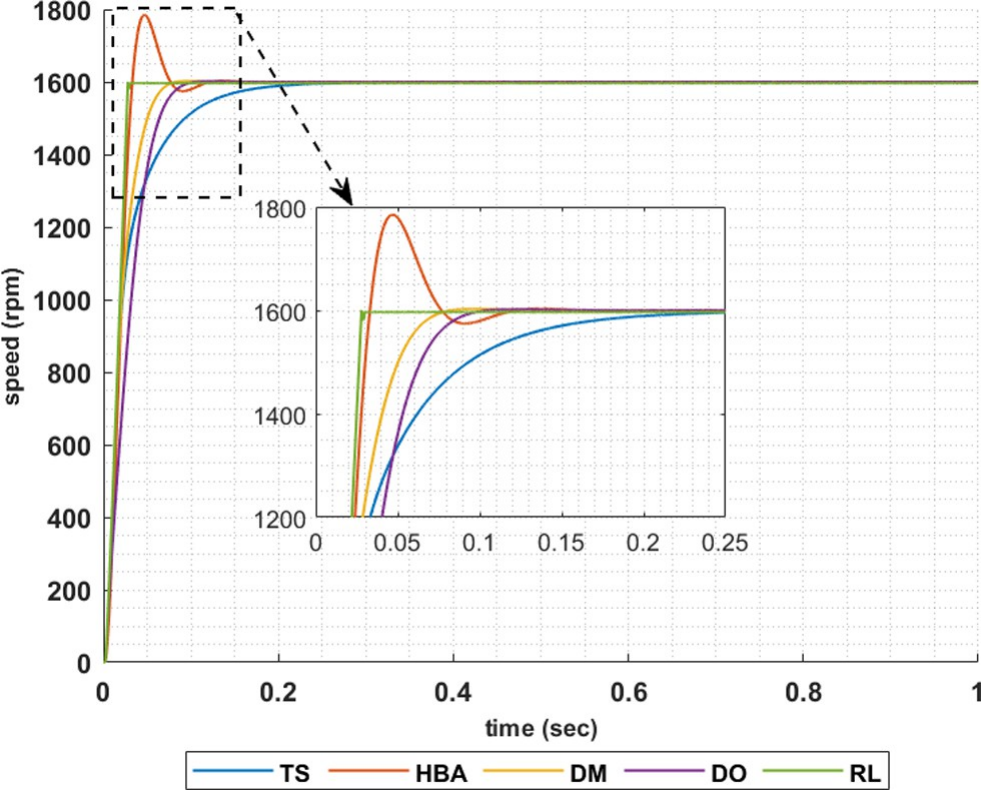
The motor speed responces when ITSE error criteria is used.

The following set of results are obtained when the motor is driving a constant load torque whose value equals to the motor rated torque, 63.662 Nm, and the reference speed is 1600 rpm. In this operating condition the motor is operated at maximum torque per amper. Figs 7–10 show the speed responses of the 5ph-IPMSM when the PI controllers gaines are obtained using the metahuristic optimization techniques (TS, HBA, DM, and DO) and the presented RL based TD3 algorithm when the four types of error critera (IAE, ISE, ITAE and ITSE) are used in the optimization techniques respectively. From these figures it can be noticed that the speed response of the 5ph-IPMSM when the presented RL based TD3 algorithm is used, to obtain PI controllers gain, has the fastes time responce and relatively low overshoot.

Table 3 summarizes the values of the PI controllers gains, settling time, rise time, maximium time and overshoot percentage for the different speed responces shown in Figs 7–10 obtained using the presented RL based TD3 algorithm and the different optimization techniques with the different error criteria. From this table, it can be noniced that the suggested RL based TD3 results in the minimum settling time and relatively low values for the rise time, max time and overshoot percentage. This proves the correctness of the suggested control methodology in improving the motor 5p-IPMSM speed response in the constant torque region.

**Table 3:** Summary of the comparison between the RL based TD3 and the metahuristic optimization techniques at speed 1600 rpm.

Technique	Error criteria	kp1	ki1	kp2	ki2	Settling Time (s)	Rise Time(s)	Max. time (s)	Overshoot Percentage
Transit Search	IAE	0.323	76.0253	149.504	108.5812	0.0723	0.0222	0.048	11.7962
	ISE	0.7304	18.9004	11.908	0.362	0.3476	0.0688	0.5302	0.0888
	ITSE	0.6456	19.8709	31.5824	9.1463	0.1459	0.067	0.5245	**0.0121**
	ITAE	0.4315	52.7312	23.4413	22.9367	0.0729	0.0266	0.0552	3.7238
Honey Badger Algorithm	IAE	0.309237	44.0829	41.9761	181.3634	0.1022	0.0364	0.0775	4.4114
	ISE	0.441908	34.8417	51.007	0	0.073	0.0443	0.1284	0.0631
	ITSE	0.335703	74.6599	170.868	60.38459	0.0706	**0.0217**	0.0468	11.4977
	ITAE	0.505046	27.6241	10.3486	25.0843	0.0954	0.0527	0.5336	0.0205
Dwarf Mongoose	IAE	0.40047	61.941	125.741	291.6633	0.0657	0.0226	0.0512	3.476
	ISE	0.641088	26.9701	33.325	57.3394	0.1222	0.0229	**0.0273**	0.5449
	ITSE	0.441913	41.2805	141.442	193.1191	0.0626	0.0385	0.096	0.2092
	ITAE	0.449873	47.7301	98.6211	8.21241	0.0439	0.029	1.7479	0.0631
Dandelion-Optimizer	ISE	0.549676	21.9602	14.511	37.1645	0.1405	0.0708	0.596	0.0164
	IAE	0.413013	37.3398	127.584	5.874622	0.065	0.0412	0.0941	0.476
	ITSE	0.392725	34.0375	103.825	146.0474	0.0787	0.0507	0.1245	0.1395
	ITAE	0.345308	42.0511	143.562	143.2322	0.0901	0.04	0.0813	2.2936
Reinforcement Learning		0.429039	44.9862	622.213	84.75749	**0.0201**	0.0274	0.028	0.0272

**Fig 11:**
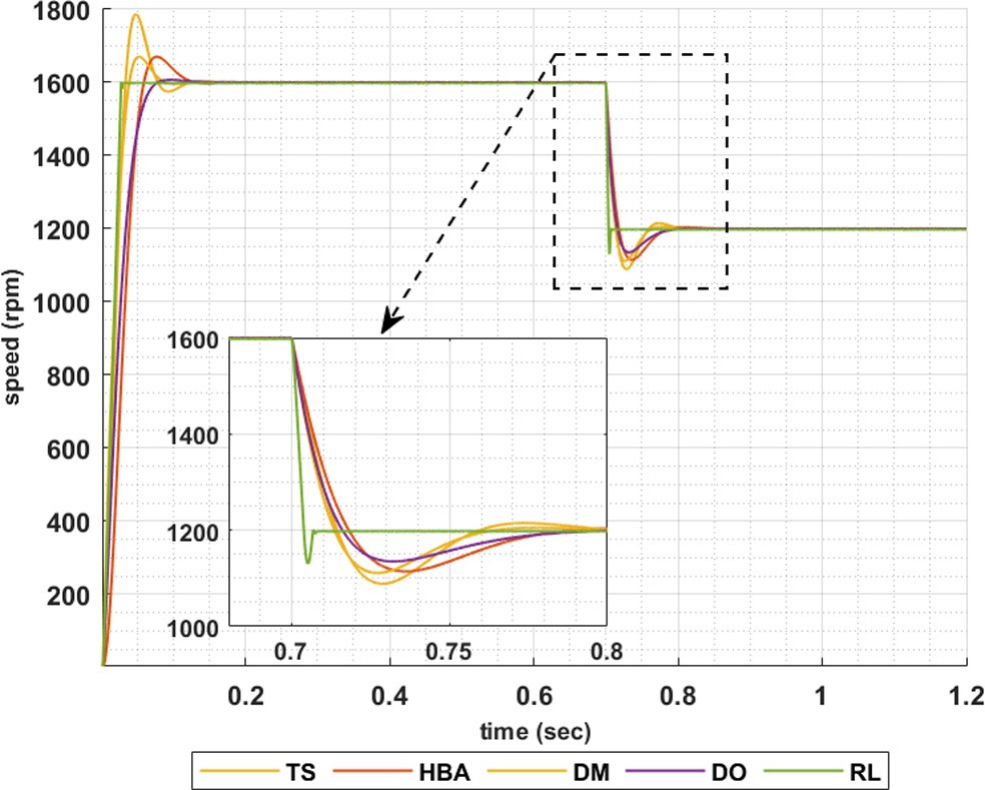
The motor speed responces when IAE error criteria is used.

**Fig 12:**
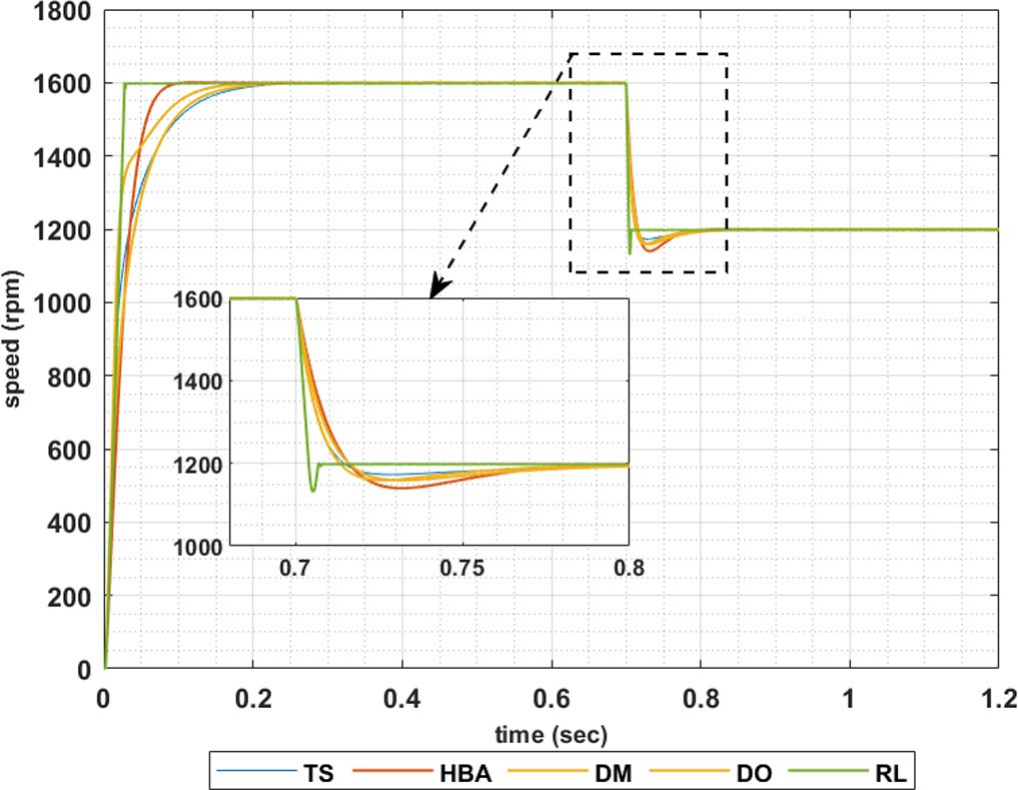
The motor speed responces when ISE error criteria is used.

**Fig 13:**
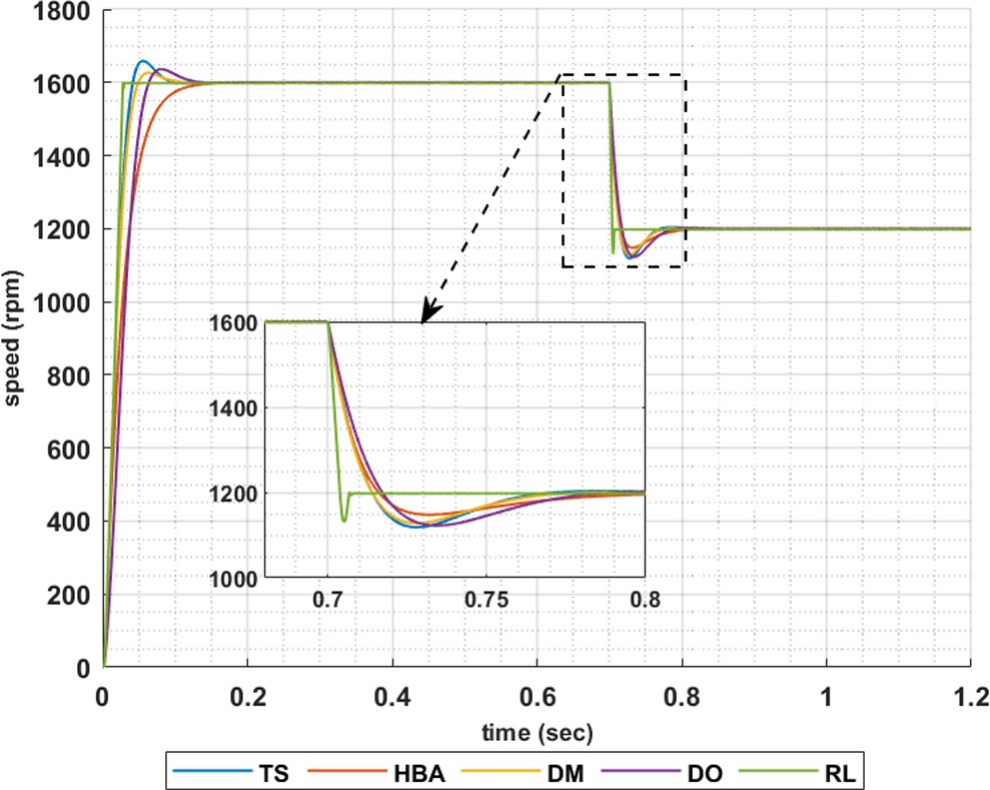
The motor speed responces when ITAE error criteria is used.

**Fig 14:**
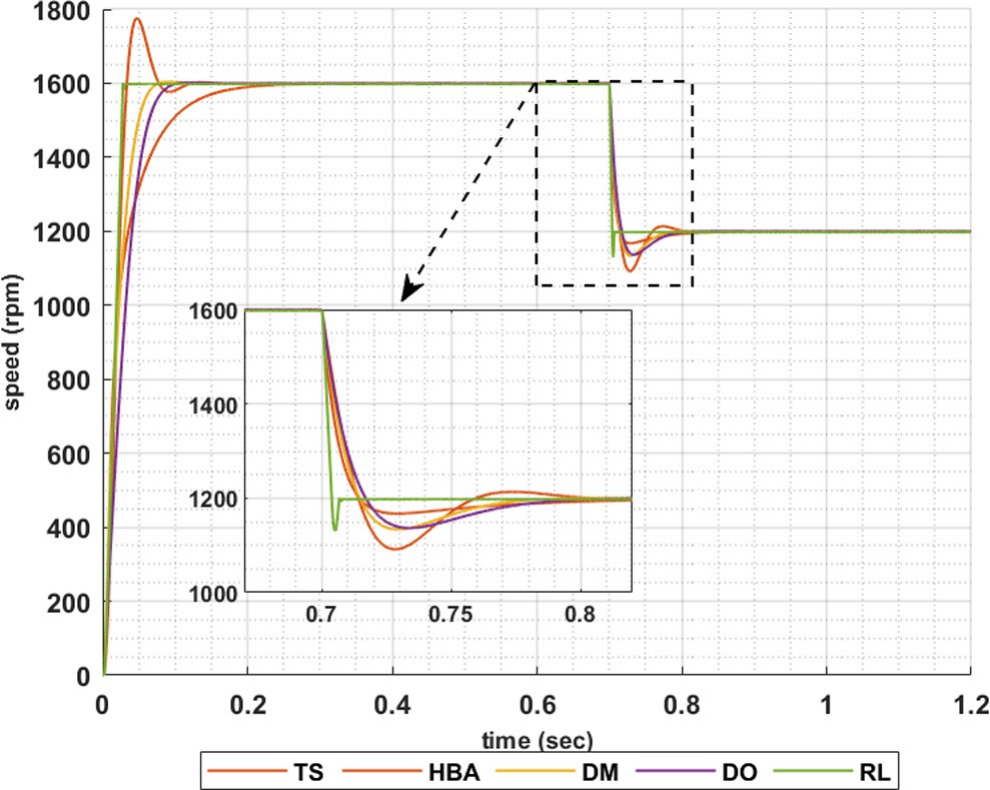
The motor speed responces when ITSE error criteria is used.

To show the correctness of the presented control methodology in case of sudden change in the reference speed, the above results are obtained when the desired speed is suddenly varied from 1600 rpm to 1200 rpm. Figs 11–14 show the speed responses for the sudden change in the motor reference speed using the metahuristic optimization techniques and the presented RL based TD3 algorithm for the four types of error critera. From these figures it can be noticed that the speed response of the 5ph-IPMSM when the presented RL based TD3 algorithm is used, also, has the fastes time responce and relatively low overshoot.

**Fig 15:**
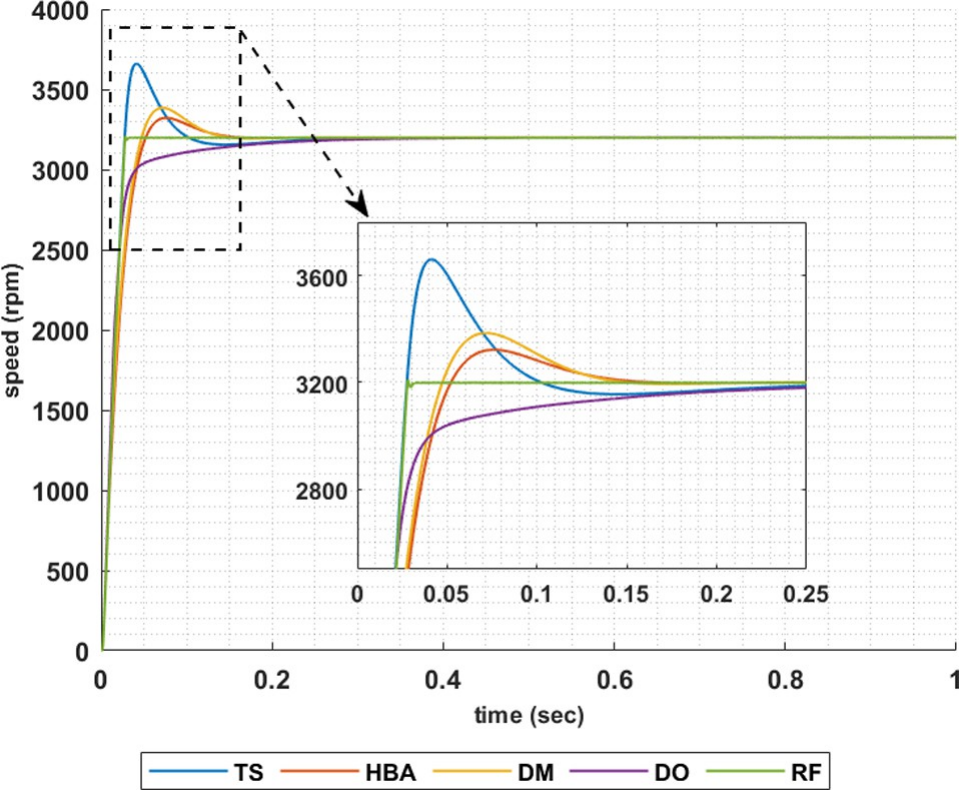
The motor speed responces when IAE error criteria is used.

**Fig 16:**
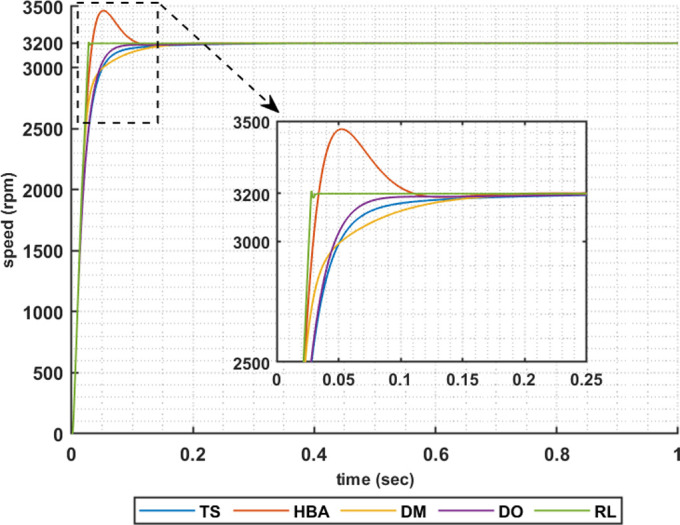
The motor speed responces when ISE error criteria is used.

**Fig 17:**
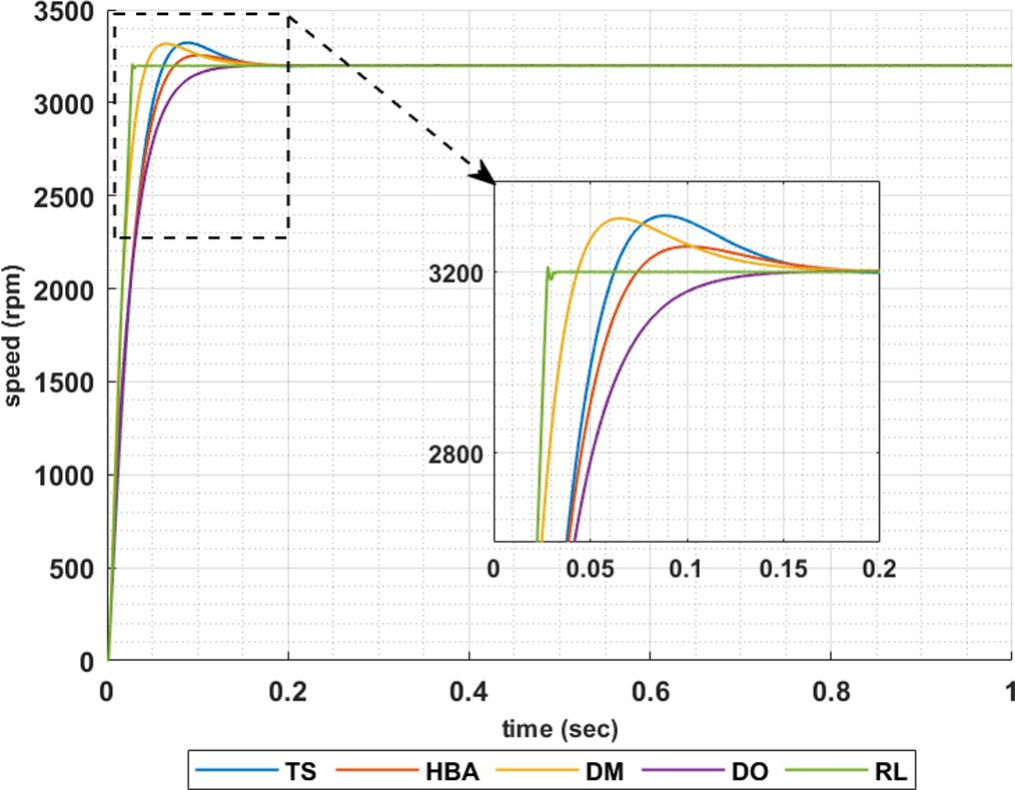
The motor speed responces when ITAE error criteria is used.

**Fig 18:**
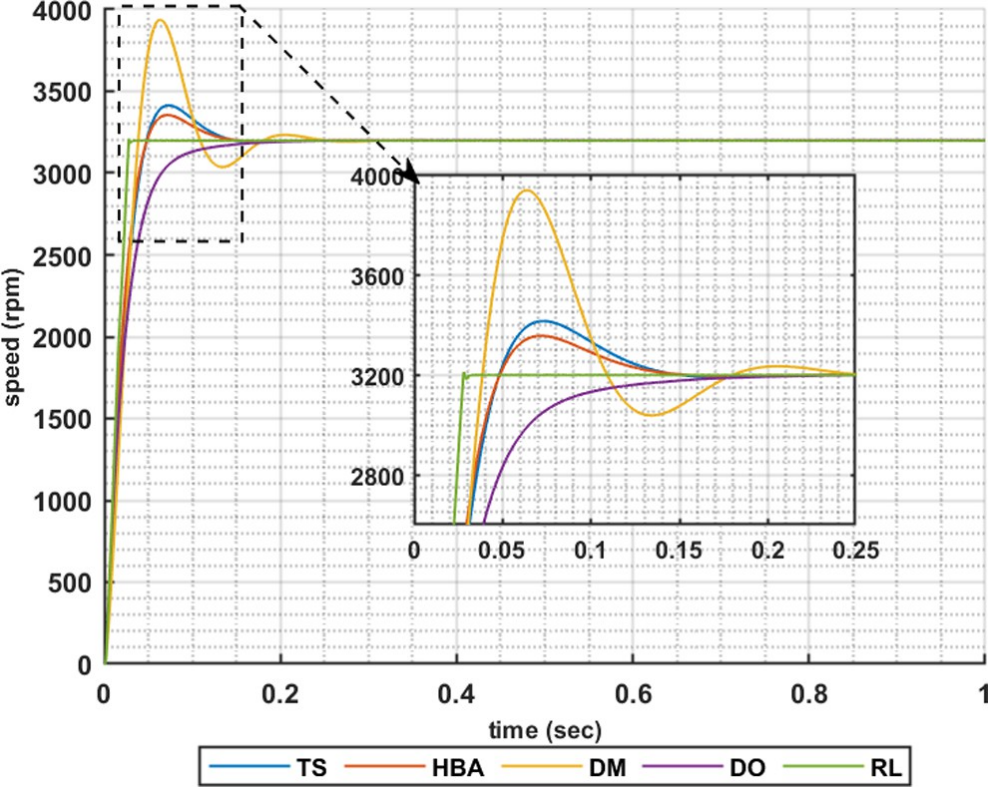
The motor speed responces when ITSE error criteria is used.

Another set of results are obtained when the motor is driving a constant load torque whose value equals to 35.8 Nm, and the reference speed is 3200 rpm. In this operating condition the motor is operated in field weakening mode of operation, i.e constant power region. Figs 15–18 show the speed responses of the 5ph-IPMSM when the PI controllers gaines are given using the metahuristic optimization techniques and the presented RL based TD3 approach when the four types of error critera are used in the optimization techniques. From these figures it can be noticed that the speed response of the 5ph-IPMSM when the presented RL based TD3 algorithm is used, to obtain PI controllers gain, has the fastes time responce and relatively low overshoot.

Table 4 summarizes the values of the PI controllers gains, settling time, rise time, maximium time and overshoot percentage for the different speed responces shown in Figs 15–18 obtained using the presented RL based TD3 algorithm and the different optimization techniques with the different error criteria when the motor is operated in constant power region. It can be noniced from Table 4 that the proposed RL based TD3 gives the minimum values for the settling time and maximum time and relatively low values for the rise time and overshoot percentage. This again proves the correctness of the suggested control methodology in improving the motor 5p-IPMSM speed response in the constant power region.

**Table 4:** Summary of the comparison between the RL based TD3 and the metahuristic optimization techniques at speed 3200 rpm.

Technique	Error criteria	kp1	ki1	kp2	ki2	Settling Time (s)	Rise Time(s)	Max. time (s)	Overshoot Percentage
Transit Search	IAE	0.5163	20.3512	74.0624	21.8978	0.0694	**0.0171**	0.0392	6.1898
	ISE	0.3243	15.5619	28.2828	10.0919	0.094	0.0406	0.5532	0.0165
	ITSE	0.2319	29.2639	200	15.4351	0.1177	0.0332	0.0737	6.7256
	ITAE	0.2225	23.2968	11.1122	51.3965	0.1232	0.0412	0.089	3.7255
Honey Badger Algorithm	IAE	0.24717	26.27137	152.6986	40.78611	0.1084	0.0336	0.0761	3.8731
	ISE	0.28545	31.4349	49.3307	49.5886	0.094	0.0247	0.0582	6.0532
	ITSE	0.24746	27.6937	40.4038	47.3758	0.1093	0.0322	0.073	4.816
	ITAE	0.23101	20.807	40.3527	24.0813	0.0665	0.0446	0.1022	1.7359
Dwarf Mongoose	IAE	0.23817	30.74569	233.662	159.5167	0.1128	0.0319	0.072	5.829
	ISE	0.31232	17.13106	105.5177	128.8104	0.0983	0.0403	0.5237	0.0082
	ITSE	0.16397	54.8639	39.9551	24.7158	0.1621	0.0279	0.0633	22.6599
	ITAE	0.32712	24.3881	61.3568	1.64838	0.0895	0.0274	0.0687	2.6998
Dandelion-Optimizer	IAE	0.55772	9.49771	107.7051	0.03886	0.1657	0.0276	0.5936	**0**
	ISE	0.3086	17.4093	42.1558	13.2945	0.0732	0.038	0.5829	0.0097
	ITSE	0.26063	16.2196	9.54845	59.3708	0.104	0.0494	0.4713	0.0091
	ITAE	0.26049	16.0258	17.9417	10.0006	0.0941	0.0524	0.2483	0.0445
Reinforcement Learning		0.20445	14.5061	88.9718	20.7561	**0.0272**	0.0205	**0.0281**	0.329

**Fig 19:**
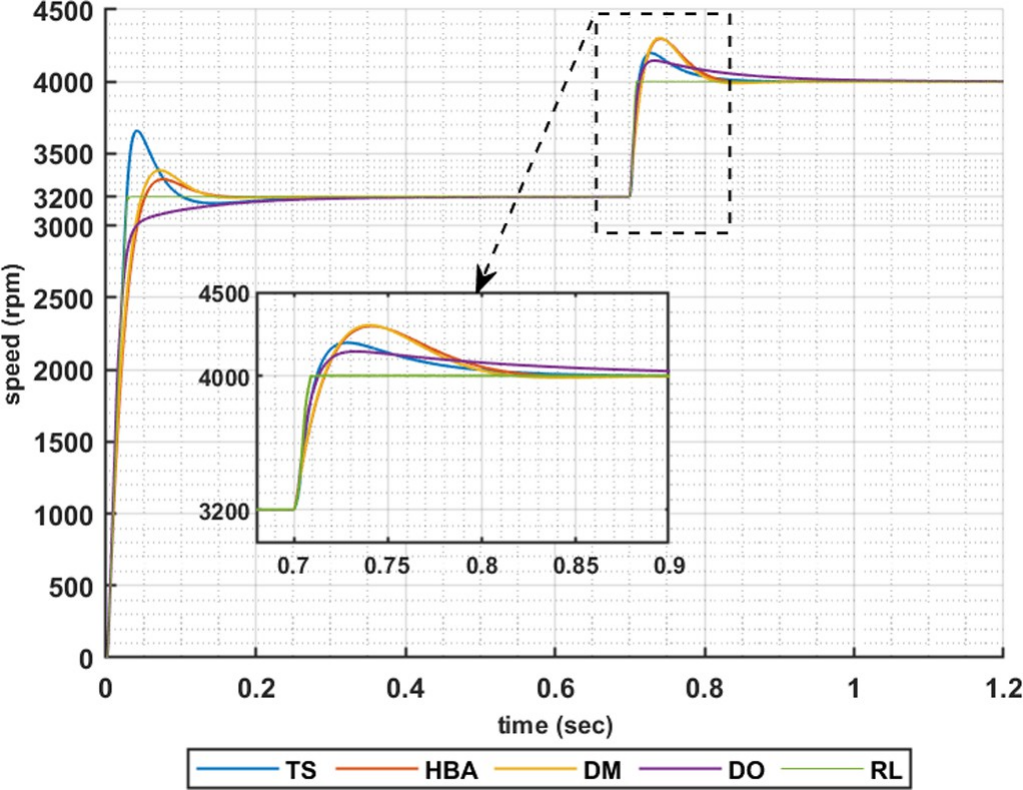
The motor speed responces when IAE error criteria is used.

**Fig 20:**
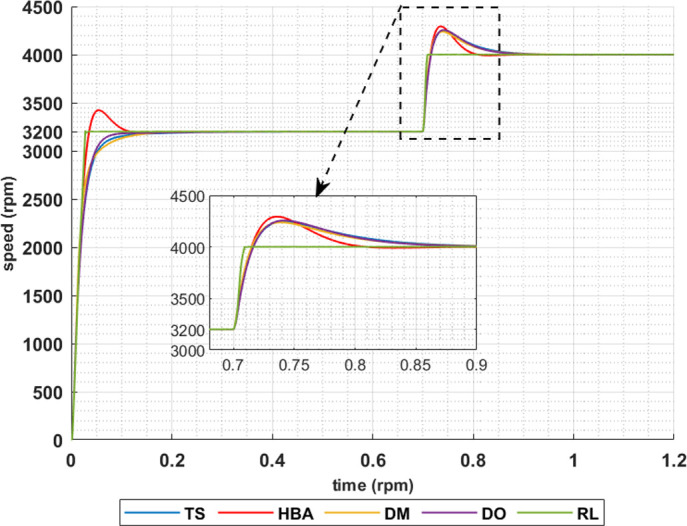
The motor speed responces when ISE error criteria is used.

**Fig 21:**
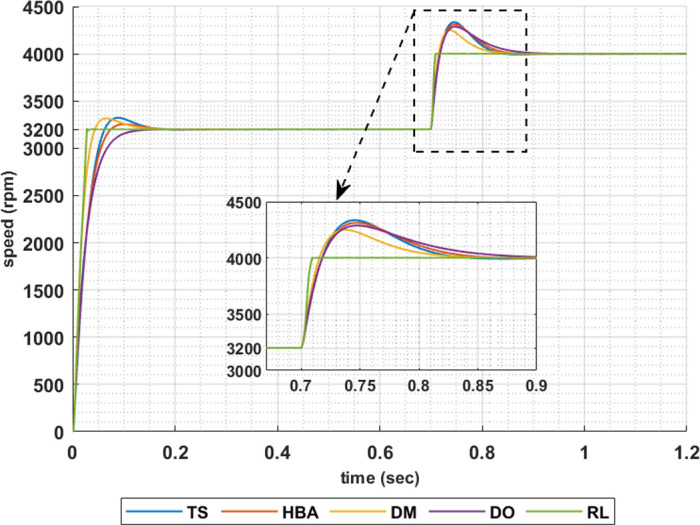
The motor speed responces when ITAE error criteria is used.

**Fig 22:**
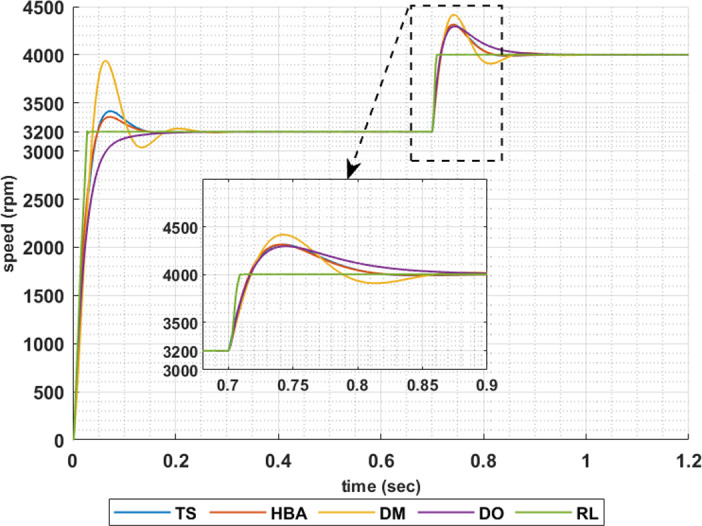
The motor speed responces when ITSE error criteria is used.

The above results are obtained when the desired speed is suddenly varied from 3200 rpm at 35 Nm load torque to 4000 rpm at 28.6 Nm load torque, constant power region. Figs 19–22 show the speed responses for the sudden change in the motor reference speed with the corresponding load torque utilizing the metahuristic optimization techniques and the presented RL based TD3 algorithm for the four types of error critera. From these figures it can be noticed that the speed response of the 5ph-IPMSM when the presented RL based TD3 algorithm is used, also, has the superior speed responce.

## 8. Conclusion

In this study, a newl RL control algorithm based TD3 algorithm is suggested to obtain gains of two cascaded PI controllers in a 5ph-IPMSM drive system. The purpose of this algorithm is to optimize the 5ph-IPMSM speed response either in constant torque region or constant power region. The PI controllers gains are also obtained using four recent metahuristic optimization techniques to be compared with the proposed algorithm. The most recent metaheuristic optimization techniques used are Transit Search, Honey Badger Algorithm, Dwarf Mongoose, and Dandelion-Optimizer optimization techniques. MATLAB Simulink package is utilized to obtain simulation results to validate the propsed algorithm. It can be concluded from the results that the suggested control RL algorithm based TD3 algorithm results in improved motor speed response compared with the metahuristic optimization techniques with the fastest response and with relatively lower overshoot either when the 5ph-IPMSM is operated either in the constant torque region or the constant power region. However, the MHOTs are more easily to be implemented and have more simple computations compared with the RL. As a future work, the experimental implementation of the proposed methodology to be achieved. In addition to this, the proposed control methodology of the drive system can be investigated with the utilization in the electric veihicles, robotics and renewable energy systems.
